# Emotional and Psychosocial Correlates of Problematic Social Media Use Among Adults: Cross-Sectional Study

**DOI:** 10.2196/82098

**Published:** 2026-04-01

**Authors:** Alexandre Hudon, Émilie Patterson, Maxime Dagenais, Saskia Cadeau, Chloé Baillargeon, Marisa Lam, Alie Le Bel, Laurie-Ann Audet, Loucie Hartal, Élodie Latreille

**Affiliations:** 1 Department of Psychiatry and Addictology Faculty of Medicine Université de Montréal Montreal, QC Canada; 2 Department of Psychiatry Institut universitaire en santé mentale de Montréal Montreal, QC Canada; 3 Centre de recherche de l'Institut universitaire en santé mentale de Montréal Montreal, QC Canada; 4 Department of Psychiatry Institut national de psychiatrie légale Philippe-Pinel Montreal, QC Canada; 5 Centre de recherche en pédagogie de la santé Montreal, QC Canada; 6 Département de médecine Faculté de médecine Université Laval Québec, QC Canada

**Keywords:** affective avoidance, digital mental health, digital stress, emotional fatigue, interface design, mental health, online behavior, problematic social media use, psychology, psychosocial correlates

## Abstract

**Background:**

Social media platforms have become integral to daily life, particularly among younger users. While they offer opportunities for connection, they also introduce new psychological stressors. Prior research has often relied on simplistic metrics such as screen time, failing to capture complex emotional and behavioral dimensions of digital engagement. There is a growing need to understand how design features and user experiences contribute to problematic social media use (PSMU), especially in adult populations.

**Objective:**

This study aims to assess the psychosocial dimensions of social media use and their associations with problematic use in an adult population, with particular attention to emotional fatigue, avoidance, and interface-induced stress.

**Methods:**

A cross-sectional online survey was completed by 402 participants, of whom 393 completed the entire questionnaire (response rate 97.8%). Recruitment was conducted through targeted advertisements on major social media platforms. Participants self-reported demographic information and completed a modified version of the CAGE-AID (Cut down, Annoyed, Guilty, Eye-opener–Adapted to Include Drugs) screener, adapted to detect PSMU. They also responded to 49 Likert scale items measuring 7 thematic psychosocial dimensions: empathic fatigue, silent stressors, identity fragmentation, pressure for visibility, algorithmic influence, digital detox behaviors, and nostalgia-linked affective responses. Descriptive and correlational statistics were used to analyze data. CAGE-AID positivity was defined as 1 or more affirmative responses.

**Results:**

Among 393 respondents (mean age 32.7, SD 10.8 years; 257/393, 65.4% women), younger age was significantly associated with PSMU as measured by modified CAGE-AID positivity (*χ*²_5_=27.0; *P*<.001; Spearman ρ=−0.160; *P*<.001). Lower education level (*χ*²_4_=11.6; *P*=.02) and employment status (*χ*²_6_=13.5; *P*=.04) were also significantly associated with PSMU. Emotional fatigue items, including reduced empathy following online emotional exposure, showed moderate correlations with PSMU (ρ=0.19-0.22; all *P*<.001). Silent interface stressors, particularly pressure to respond due to “seen” indicators, were positively associated with PSMU (ρ=0.124-0.153; *P*=.013). Online identity curation demonstrated the strongest association (ρ=0.280; *P*<.001). Digital detox behaviors, including guilt and sleep disruption, were also significantly correlated with PSMU (ρ=187-0.200; *P*<.001).

**Conclusions:**

PSMU in adults is closely tied to emotional fatigue, interface-related stress, and avoidance. Younger users appear particularly susceptible to emotional saturation and compulsive engagement. These findings highlight the need to consider psychological and design-related mechanisms in public health responses to digital overuse. Interventions should move beyond screen time to address emotional reactivity and structural stressors embedded in platform design.

## Introduction

Social media has emerged as a dominant force in contemporary digital culture, reshaping communication, identity formation, and emotional expression. As of 2023, over 60% of the global population actively uses social media, with daily usage averaging more than 2 hours per person [1,2]. Platforms like Instagram, TikTok, Facebook, Snapchat, and X (formerly known as Twitter) are not merely channels for information and entertainment: they have become integral to how people present themselves, maintain relationships, and engage with the world [3]. These digital spaces operate on feedback loops that amplify emotional content and favor visibility, governed by algorithms that personalize content based on user behavior [4]. As users become increasingly immersed in these environments, the psychological dynamics of social interaction, social comparison, and impression management have shifted from offline to online domains, introducing new cognitive and affective demands [5,6].

Literature increasingly demonstrates that social media use is associated with both salutary and adverse mental health outcomes, with effects varying according to context of use, platform architecture, and individual vulnerability profiles [7-9]. On one hand, digital environments may facilitate social connectedness, identity exploration, community belonging, and rapid access to health-related information. On the other, a substantial body of evidence links heavy or maladaptive engagement to elevated symptoms of depression, anxiety, sleep disruption, diminished self-esteem, and broader psychological distress [10-13]. Importantly, these associations are not merely functions of time spent online but appear to be mediated by specific psychosocial mechanisms. Upward social comparison processes, repeated exposure to emotionally arousing or polarizing content shaped by algorithmic amplification, and fear of missing out have all been identified as pathways through which digital engagement may exacerbate emotional vulnerability [14,15].

Beyond these mechanisms, emerging constructs further underscore the complexity of digital mental health dynamics. Prolonged exposure to emotionally charged or traumatic content may contribute to emotional exhaustion and desensitization, sometimes conceptualized as “digital empathic fatigue,” reflecting a form of affective overload unique to high-volume online environments [16]. Concurrently, the sustained management of one’s online persona (balancing authenticity, audience expectations, and performance metrics) can generate chronic self-monitoring and identity strain [17]. Together, these findings suggest that the psychological impact of social media is best understood as multidimensional, shaped by interacting emotional, cognitive, and structural features of digital platforms rather than by exposure alone.

Despite increasing interest in the psychological consequences of social media use, the literature still faces several limitations. Many studies rely on simplistic measures such as screen time or general frequency of use, which fail to capture the complexity of users’ emotional and cognitive experiences online [18,19]. The specific effects of platform design features (such as algorithmic feeds, read receipts, viral metrics, or digital memory functions) remain underexplored, even though these features may subtly reinforce compulsive behavior or emotional dysregulation [20-22]. Furthermore, most existing research has been conducted in adolescent samples, with less attention to adult populations who may experience different forms of psychosocial stress in digital environments [23,24]. There is a pressing need for more multidimensional analyses that account for the psychological nuances of digital engagement and move beyond correlational metrics to consider the lived experiences and emerging stressors associated with social media.

The primary objective of this study is to explore the perceived effects of social media use on the mental health of adult users by analyzing quantitative data from thematic scales. Through this approach, the study attempts to better understand how specific platform features (such as algorithms, viral content dynamics, memory notifications, and read receipts) may influence psychological well-being. In parallel, this study aims to assess the intensity of various psychosocial dimensions linked to social media use, identify user profiles at greater risk of emotional distress based on sociodemographic and behavioral variables, and uncover recurring themes within participants’ lived experiences. This research intends to provide a more nuanced and integrated understanding of how digital practices intersect with mental health. Finally, we aim to highlight the mechanisms underlying social media–related distress and to inform more targeted strategies for prevention and intervention.

## Methods

### Ethical Considerations

This study was approved by the Research Ethics Committee in Education and Psychology of the Université de Montréal (approval number 2025-6867). Participants were presented with an information letter outlining the purpose and procedures of the study and were required to provide informed consent prior to participation. All data were collected anonymously, and no identifying information was retained. Participants did not receive any form of compensation for their involvement in the study.

### Study Design

This study used a cross-sectional design using a survey approach. Data were collected through an online survey created and administered via LimeSurvey, accessible in both French and English. The survey aimed to assess psychosocial factors associated with social media use in the general adult population. All responses were anonymous and collected over an 8-week period. The choice of an online format was appropriate given the digital literacy of the target population and the thematic focus on social media use. We followed the STROBE (Strengthening the Reporting of Observational Studies in Epidemiology) checklist (Multimedia Appendix 1) to strengthen the reporting of this observational cross-sectional study [25].

### Participants and Recruitment

Participants were recruited through organic (nonpaid) postings on popular social media platforms, including Facebook, Instagram, Reddit, and TikTok. A standardized study invitation describing a research project on social media habits and emotional experiences was publicly shared on these platforms and within selected online communities. No paid advertising or algorithmic demographic targeting was used; participation relied entirely on voluntary self-selection by individuals who encountered the post and chose to access the survey link.

Individuals who clicked the link were redirected to a secure LimeSurvey platform hosted on institutional servers. Inclusion criteria were (1) age of 18 years or older (self-attested), (2) regular use of at least 1 social media platform, and (3) provision of informed digital consent prior to participation. Exclusion criteria included reporting no social media use or submitting incomplete questionnaires. Because the survey was fully anonymous and did not collect identifying information, eligibility criteria such as age were based on participant self-report.

The target sample size ranged between 300 and 500 participants. This range was informed by comparable cross-sectional digital health surveys and supported by an a priori power analysis conducted using G*Power (version 3.1.9.7), which indicated that a minimum sample of 385 participants would provide adequate statistical power (95% confidence level, 5% margin of error) to detect small-to-moderate correlational effects [26].

### Data Collection

Sociodemographic variables collected included age, sex assigned at birth, country of residence, living environment (urban, rural, or other), current employment status, highest level of education attained, and estimated daily time spent on social media (in hours). These variables were selected for their relevance to previous literature on digital media use and mental health risk factors.

To assess problematic or compulsive social media use, the study adapted the CAGE-AID (Cut down, Annoyed, Guilty, Eye-opener–Adapted to Include Drugs), a validated 4-item screener commonly used for alcohol use disorders and substance abuse [27]. In this adaptation, items were reworded to target digital behaviors and emotional responses rather than substance use. For instance, questions assessed participants’ desire to reduce social media use, emotional discomfort when criticized about their usage, feelings of guilt about time spent online, and compulsive checking behavior, such as opening social media immediately upon waking. Each item had a binary response format (yes/no), and a score of 1 or more “yes” responses was interpreted as a positive screen, consistent with established usage of the original tool. The rationale for this adaptation lies in the emerging consensus that behavioral addictions, such as problematic social media use (PSMU), share features with substance use disorders (including impaired control, emotional salience, and functional impairment), making the CAGE-AID a reasonable, if preliminary, proxy for identifying at-risk individuals in population surveys [28].

Beyond the screener, participants completed a series of Likert-type items related to 7 thematic psychosocial dimensions of social media use: empathic fatigue, silent stressors (eg, read receipts and typing indicators), identity fragmentation, pressure for visibility and virality, algorithmic influence, digital detox behaviors, and nostalgia-related emotional responses. Each dimension was explored using a standardized set of 7 statements, rated on a 5-point Likert scale ranging from “strongly disagree” to “strongly agree.” These items were developed from a synthesis of emerging literature and pilot feedback and aimed to capture the subjective intensity of each experience. The full questionnaire is available in French and in English in Multimedia Appendix 2.

### Statistical Analysis

All quantitative analyses were conducted using Python (version 3.11) and the scikit-learn library [29]. Descriptive statistics were computed to characterize the sample and provide means for each psychosocial dimension. Associations between sociodemographic variables and CAGE-AID positivity were examined using chi-square tests for categorical variables and Spearman’s ρ for ordinal or nonnormally distributed continuous variables. The threshold for statistical significance was set at *P*<.05 for all tests. Data were screened for missingness and outliers prior to analysis, and appropriate assumptions for each test were verified.

## Results

### Sociodemographic Characteristics of the Respondents

The surveyed population was composed of a diverse sociodemographic cohort of 402 respondents, of whom 393 completed the questionnaire. Participants ranged across various age groups, with a larger representation from younger adults (particularly those aged 18-34 years). The gender distribution included both male and female respondents, with an overrepresentation of women, which is common in mental health–oriented surveys [30]. Most participants resided in urban or suburban environments, and the majority were either employed full-time or students. A high level of education was observed in the sample, with many respondents holding university degrees, including graduate-level qualifications. Daily time spent on social media varied, but several participants reported spending 3 or more hours per day, which may reflect the integration of digital platforms into daily routines. The sample skewed toward a digitally connected, educated, and professionally active population. The main characteristics of the population and correlations with the CAGE-AID are presented in Table 1.

**Table 1:** Sociodemographic characteristics and associations with problematic social media use (modified CAGE-AID [Cut down, Annoyed, Guilty, Eye-opener–Adapted to Include Drugs] ≥1) in a cross-sectional online survey of adult social media users (aged 18 years or older) recruited via organic social media postings over an 8-week period. Associations were tested using chi-square and Spearman rank correlation (ρ) analyses.

Variable and subgroup		Total, n	CAGE-AID (modified) positive, n	Positive, %	*P* value (chi-square test)	Spearman rank correlation, ρ	*P* value (Spearman rank correlation)
**Age group (years)**							
	18-24	108	104	96.3	<.001	–0.126	.01
	25-34	129	123	95.3	<.001	–0.126	.01
	35-44	58	56	96.6	<.001	–0.126	.01
	45-54	48	43	89.6	<.001	–0.126	.01
	55-64	41	38	92.7	<.001	–0.126	.01
	65 and above	9	5	55.6	<.001	–0.126	.01
**Gender**							
	Female	312	295	94.6	.49	–0.053	.30
	Male	79	72	91.1	.49	–0.053	.30
	Other	2	2	100	.49	–0.053	.30
**Country**							
	Algeria	3	3	100	>.99	–0.034	.51
	Canada	353	332	94.1	>.99	–0.034	.51
	France	14	13	92.9	>.99	–0.034	.51
	India	1	1	100	>.99	–0.034	.51
	Netherlands	2	2	100	>.99	–0.034	.51
	Norway	1	1	100	>.99	–0.034	.51
	Philippines	1	1	100	>.99	–0.034	.51
	Portugal	1	1	100	>.99	–0.034	.51
	Russia	1	1	100	>.99	–0.034	.51
	United Kingdom	2	2	100	>.99	–0.034	.51
	United States of America	14	12	85.7	>.99	–0.034	.51
**Living environment**							
	Suburb	161	148	91.9	.12	0.091	.07
	Rural	39	35	89.7	.12	0.091	.07
	Urban	193	186	96.4	.12	0.091	.07
**Employment**							
	Other	7	6	85.7	.04	0.091	.07
	Full-time	175	162	92.6	.04	0.091	.07
	Part-time	25	23	92	.04	0.091	.07
	Retired	19	15	78.9	.04	0.091	.07
	None	5	5	100	.04	0.091	.07
	Self-employed	37	37	100	.04	0.091	.07
	Student	125	121	96.8	.04	0.091	.07
**Education**							
	Other	11	11	100	.02	–0.104	.04
	Masters or Doctorate (including professional doctorates)	145	139	95.9	.02	–0.104	.04
	Bachelor’s degree	137	128	93.4	.02	–0.104	.04
	Vocational training or technical degree	69	66	95.7	.02	–0.104	.04
	Highschool or less	31	25	8.6	.02	–0.104	.04
**Time on social media, hours/day**							
	1-2	143	131	91.6	.002	–0.006	.91
	3-4	176	172	97.7	<.001	–0.006	.91
	5-6	40	39	97.5	<.001	–0.006	.91
	7	10	9	90	<.001	–0.006	.91
	<1	24	18	75	<.001	–0.006	.91

### Main Findings on CAGE-AID and Sociodemographic Correlates

Analysis of the adapted CAGE-AID screener for PSMU revealed meaningful associations with several sociodemographic factors. Younger age groups demonstrated a significantly higher likelihood of screening positive on the CAGE-AID, with the highest positivity rate observed among those aged 18 to 24 years. This trend was statistically supported by both a significant chi-square result (*χ*²_5_=27.0; *P*<.001) and a negative Spearman correlation (ρ=–0.160; *P*<.001), suggesting an inverse relationship between age and problematic use. Similarly, lower levels of education were associated with increased CAGE-AID positivity, reinforcing the possibility that educational attainment may act as a protective factor (*χ*²_4_=11.6; *P*=.02; ρ=–0.121). Employment status also emerged as a relevant factor, with higher positivity rates among students and those in less stable employment (*χ*²_6_=13.5; *P*=.04). While time spent on social media showed a significant global association (*χ*²_4_=21.9; *P*<.001), the corresponding Spearman rank correlation was weak (ρ=–0.097), suggesting that the quantity of use alone may be less informative than the psychological relationship with these platforms. No significant associations were observed for gender, country of residence, or living environment, indicating these variables may not independently predict PSMU in this sample.

### Fatigue Empathy Linked to Social Media Usage

**Figure 1:**
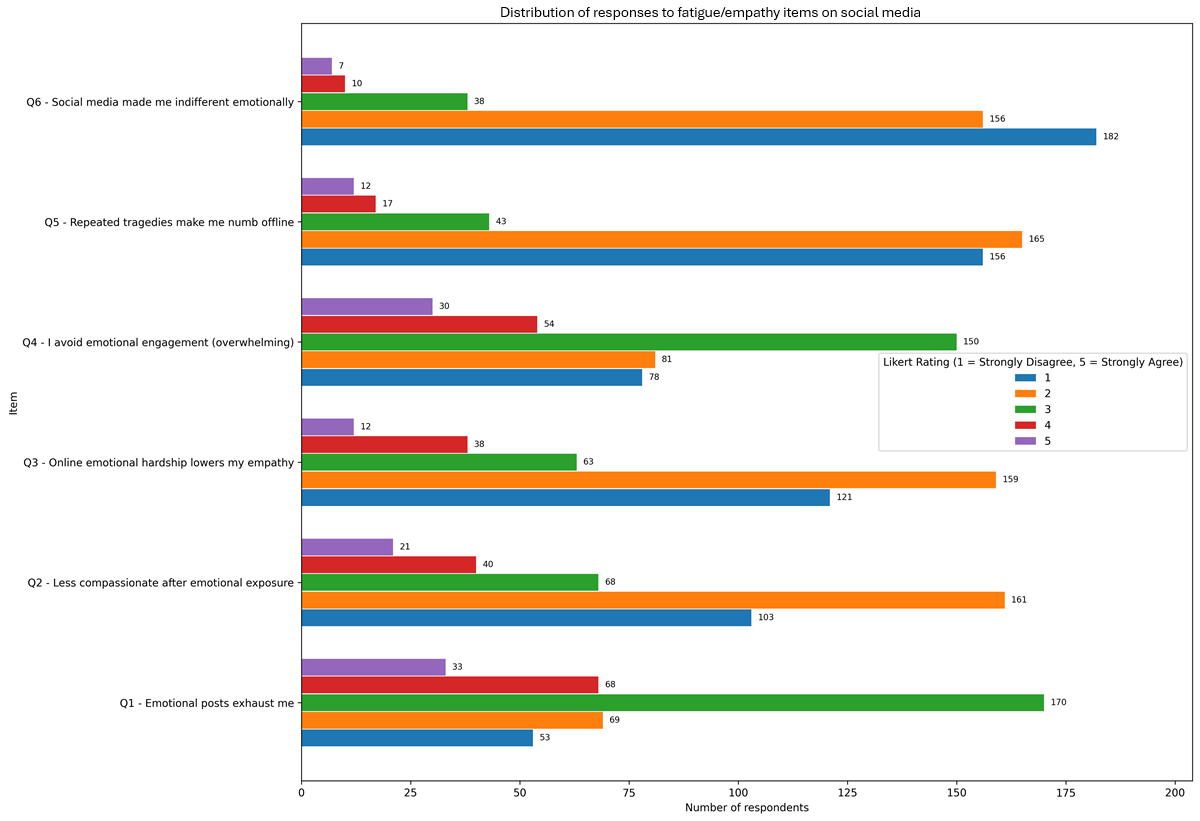
Mean Likert scale endorsement (1-5) of emotional fatigue and empathy-related experiences among adult social media users in a cross-sectional online survey conducted over 8 weeks. Associations with problematic social media use were examined using Spearman rank correlations.

Emotional fatigue and empathy erosion in relation to prolonged social media use responses are presented in Figure 1. Mean scores indicate that the most endorsed items were “Emotional posts exhaust me” mean 3.35 (SD 1.10) and “I avoid emotional engagement because it’s overwhelming” mean 3.20 (SD 1.12), highlighting a prevalent sense of emotional saturation online. Conversely, statements reflecting emotional detachment, such as “Social media made me indifferent to emotional hardship” mean 2.30 (SD 0.77), were less strongly endorsed.

In terms of correlations with PSMU (defined as 1 or more positive responses on the adapted CAGE-AID), the strongest association emerged for the item “Online emotional hardship lowers my empathy” (ρ=0.22; *P*<.001), suggesting a potential link between emotional desensitization and maladaptive usage patterns. Other items such as “Less compassionate after emotional exposure” and “I avoid emotional engagement” also showed significant positive correlations with PSMU (ρ=0.20; *P*<.001 and ρ=0.19; *P*<.001, respectively).

Subgroup analyses revealed that younger age groups, particularly those aged 18 to 24 years, tended to report higher agreement with emotionally fatiguing statements, with scores gradually decreasing in older cohorts. This suggests that emotional saturation and disengagement may disproportionately affect younger users, who are also more likely to be heavy social media users and more susceptible to CAGE-AID positivity.

### Psychosocial Impact of Silent Factors in Social Media

**Figure 2:**
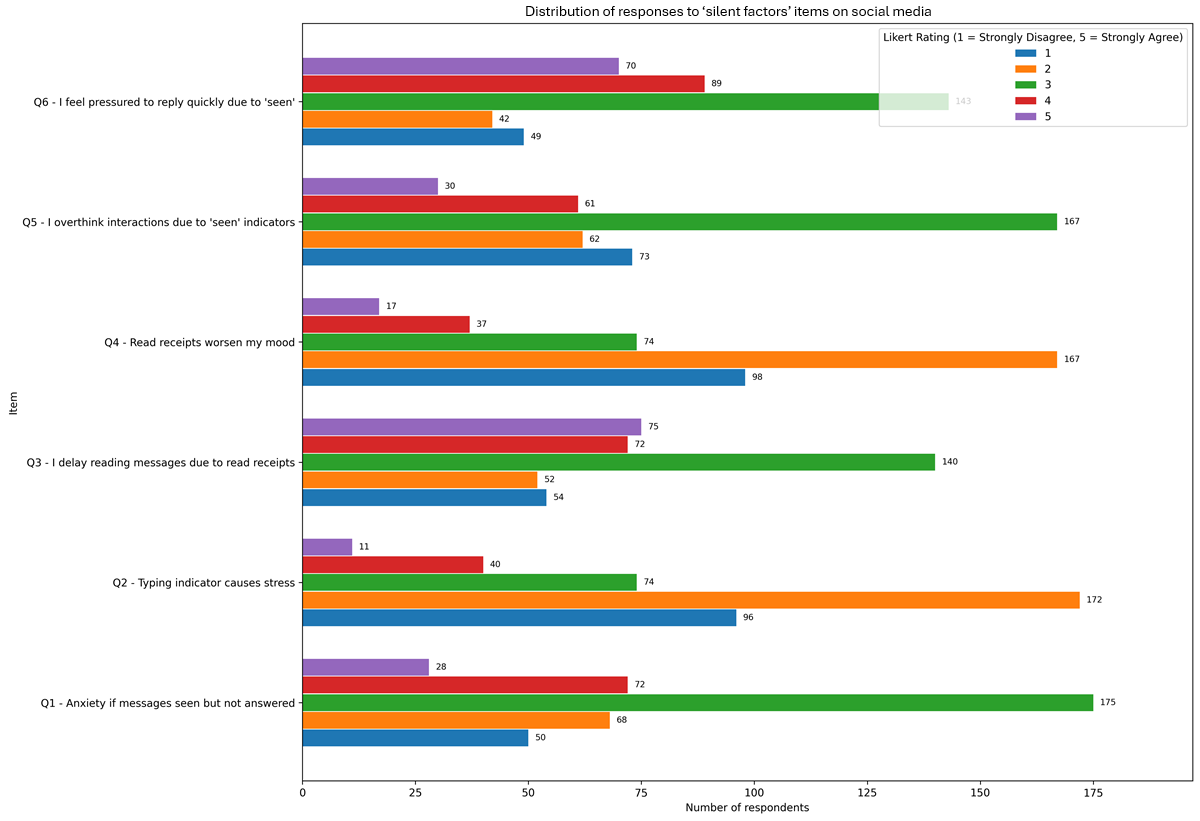
Mean endorsement (1-5 Likert scale) of stress related to messaging interface features (eg, read receipts and typing indicators) in adult social media users from a cross-sectional online survey (8-week recruitment period).

The “silent factors” explored in this survey (such as read receipts, typing indicators, and perceived delays in replies) emerge as subtle but significant sources of digital stress. Responses of the respondents are presented in Figure 2. The items most frequently endorsed were “I feel pressured to reply quickly due to ‘seen’ indicators” mean 3.44 (SD 1.08) and “I delay reading messages due to read receipts” mean 3.16 (SD 1.12), suggesting that interface cues in messaging apps may generate a sense of urgency and avoidance behaviors. In contrast, items like “Typing indicators cause me stress” mean 2.23 (SD 1.05) and “Read receipts worsen my mood” mean 2.26 (SD 1.03) were endorsed less frequently, indicating more individualized reactions to these features.

Correlational analyses showed that several items had modest but statistically significant associations with CAGE-AID positivity, indicating potential links between digital communication stress and PSMU. Specifically, “I delay reading messages due to read receipts” and “I overthink interactions due to ‘seen’ indicators” both correlated significantly with CAGE-AID scores (ρ=0.153 and ρ=0.146, respectively; *P*<.001), while “Feeling pressured to reply quickly” also showed a positive association (ρ=0.124; *P*=.01). These findings suggest that users who internalize social expectations embedded in app features may be more likely to report problematic patterns of use.

Subgroup analysis by age revealed that younger respondents reported higher levels of stress and reactivity across nearly all silent factor items. For example, participants aged 18 to 24 years averaged a higher agreement with statements as “I feel anxious if messages are seen but not answered” mean 2.90 (SD 1.07) and “I overthink interactions due to ‘seen’ indicators” mean 2.78 (SD 1.05). This pattern reinforces prior observations that digital social stressors disproportionately affect younger users, possibly due to greater online social dependence, peer validation needs, and digital communication intensity. Therefore, these results highlight that messaging app design may unintentionally contribute to psychological strain, especially in vulnerable populations.

### Online Identity Fragmentation and Its Psychological Implications

**Figure 3:**
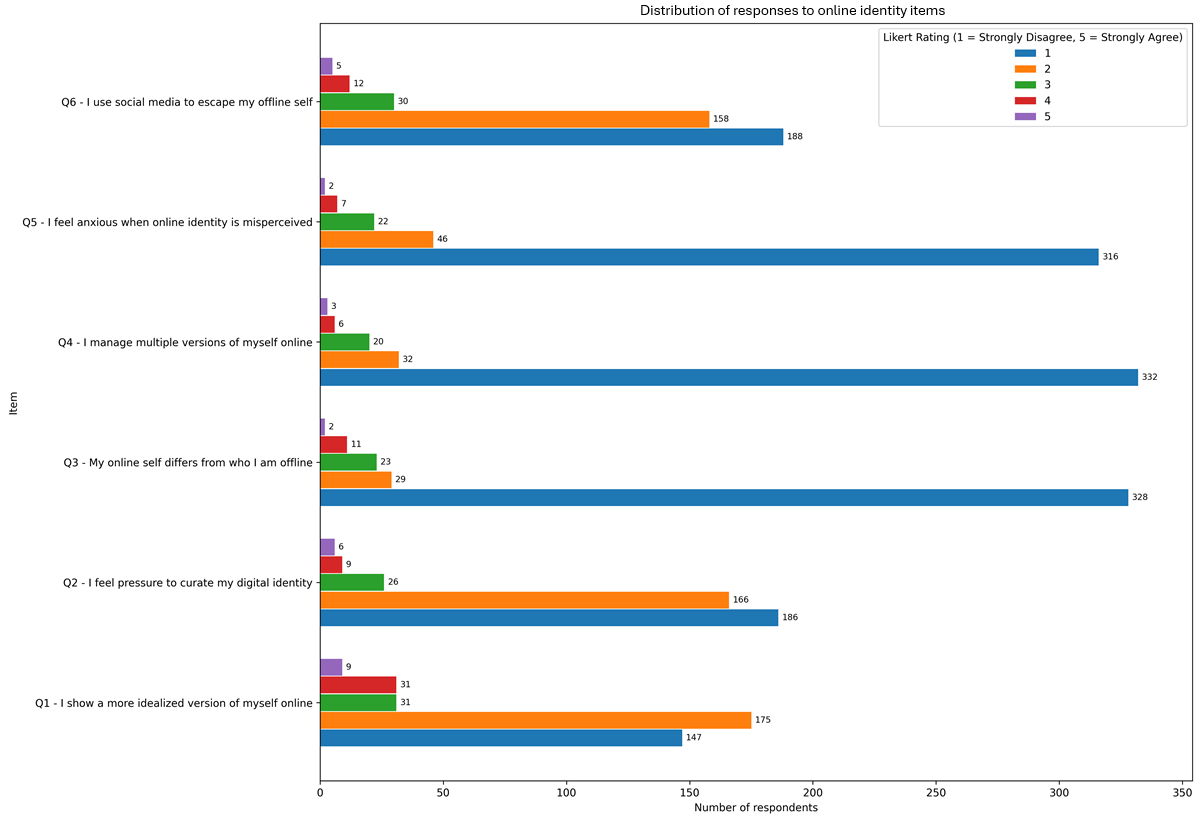
Mean endorsement (1-5 Likert scale) of online identity–related pressures (eg, idealization and curation) among adult social media users in a cross-sectional online study conducted over 8 weeks.

Responses to the Online Identity scale reveal nuanced tensions between self-presentation and authenticity in digital spaces and are presented in Figure 3. The most endorsed items were “I show a more idealized version of myself online” mean 1.95 (SD 0.92) and “I feel pressure to curate my digital identity” mean 1.84 (SD 0.90), indicating that many participants (though not strongly) experience subtle pressures to maintain a carefully crafted online image. Other items such as “I manage multiple versions of myself online” and “I use social media to escape my offline self” had lower endorsement (means ranging from 1.2 to 1.5), suggesting that these behaviors may be more specific to certain users rather than generalized across the sample.

Correlational analyses with CAGE-AID positivity highlight a significant relationship between online identity manipulation and PSMU. The item “I feel pressure to curate my digital identity” showed the strongest correlation with CAGE-AID scores (ρ=0.280; *P*<.001), followed by “I show a more idealized version of myself online” (ρ=0.234; *P*<.001). These findings suggest that individuals who invest more in crafting or controlling their online persona may be more likely to engage in maladaptive social media behaviors. Lesser but still significant correlations were observed for items related to anxiety over misrepresentation (ρ=0.132; *P*=.009) and using social media for escapism (ρ=0.117; *P*=.02).

Subgroup analysis by age revealed a clear downward trend: younger participants (aged 18-34 years) reported higher mean scores on nearly all identity-related items compared to older groups. For example, the group of respondents aged 18 to 24 years had the highest mean for “I show a more idealized version of myself” mean 1.99 (SD 0.93) and “I feel pressure to curate” mean 1.74 (SD 0.90), whereas respondents aged 55 years or older consistently reported means below 1.5 across all items. This trend suggests that digital identity tension is especially prominent in younger adults, potentially driven by increased social media exposure, peer comparison, and developmental identity work.

### Online Viral Presence: Motivations, Validation, and Age-Sensitive Trends

**Figure 4:**
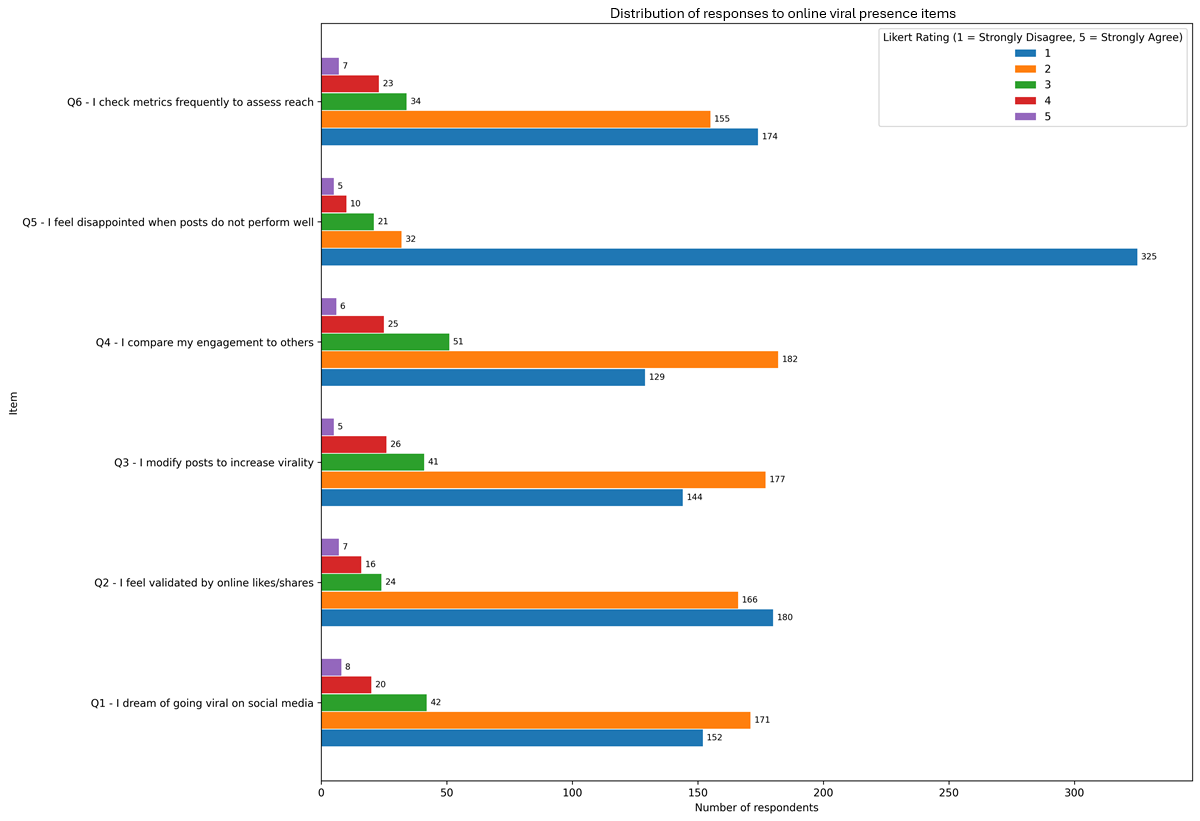
Mean endorsement (1-5 Likert scale) of perceived algorithmic influence among adult social media users in a cross-sectional online survey conducted over 8 weeks.

Participant responses on viral presence and engagement-related behaviors reveal a low-to-moderate overall endorsement, with nuanced psychological implications. They are presented in Figure 4. The most endorsed items included “I compare my engagement to others” mean 2.06 (SD 1.00) and “I dream of going viral on social media” mean 1.88 (SD 0.95), reflecting some aspirational and comparative tendencies related to online visibility. Meanwhile, items reflecting emotional reactivity such as “I feel disappointed when posts don’t perform well” mean 1.30 (SD 0.85) had the lowest mean, suggesting that for most users, personal validation is not entirely contingent on digital metrics.

Correlational analysis identified significant, though modest, associations between several viral presence items and CAGE-AID positivity. The strongest were “I compare my engagement to others” (ρ=0.134; *P*=.008) and “I modify posts to increase virality” (ρ=0.130; *P*=.01), suggesting that a performance-oriented approach to content curation may be linked to PSMU. Other items like “I feel validated by likes/shares” and “I check metrics frequently” showed weaker, nonsignificant correlations, reinforcing that not all engagement behaviors are inherently maladaptive.

Subgroup analysis by age revealed a consistent trend: younger age groups (aged 18-24 years and 25-34 years) reported higher agreement with nearly all items, particularly for “dreaming of going viral” mean 2.02 (SD 0.98) in those aged 18-24 years and “comparing engagement” mean 2.11 (SD 1.02). These ratings declined with age, reaching the lowest levels among those aged 55 years or older. This pattern underscores the generational dynamics of social media use, with younger users potentially more invested in visibility, peer comparison, and digital identity performance. The findings suggest that engagement-oriented and comparative behaviors (hallmarks of viral motivation) may serve as subtle contributors to compulsive social media habits, especially in younger populations navigating online validation pressures.

### Algorithmic Influence: Perception, Resistance, and Associations With Social Media Overuse

**Figure 5:**
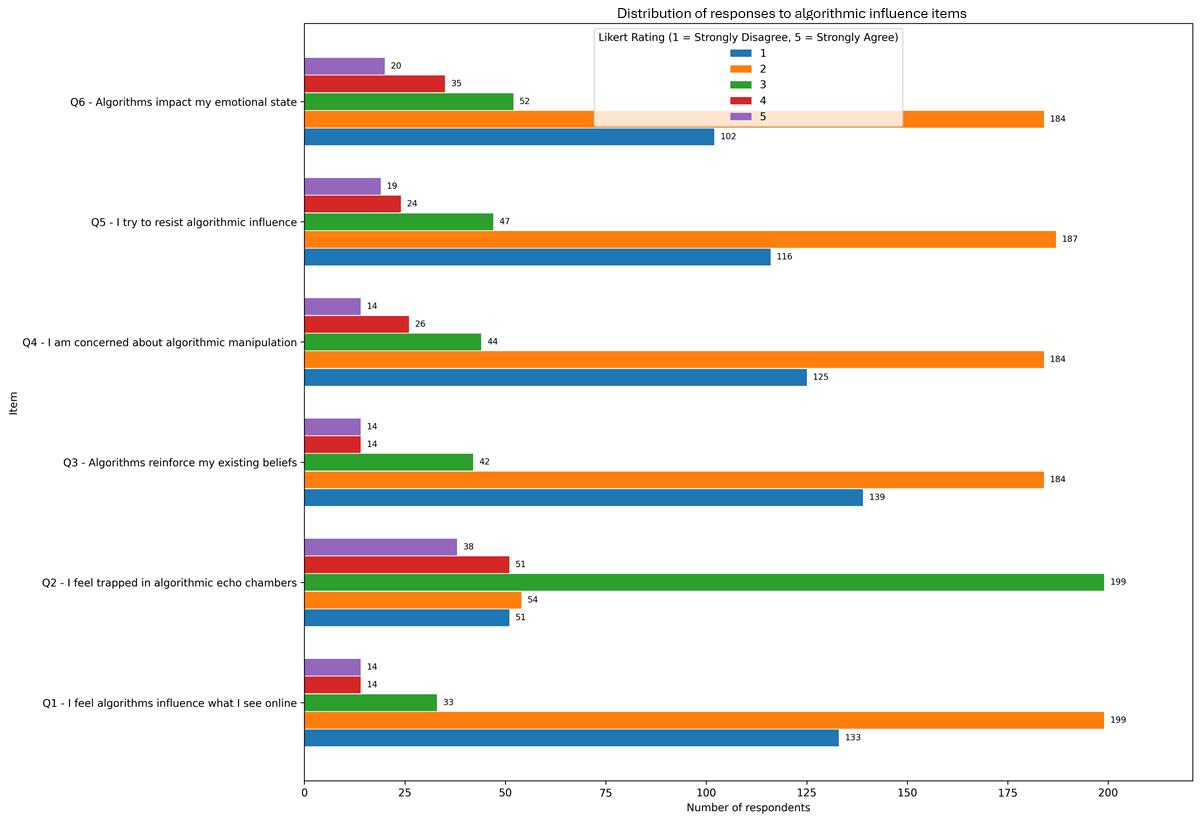
Mean endorsement (1-5 Likert scale) of perceived algorithmic influence among adult social media users in a cross-sectional online survey conducted over 8 weeks.

Participants’ responses indicate a nuanced awareness of the role algorithms play in shaping online experiences, accompanied by varying degrees of psychological impact and are presented in Figure 5. The highest rated item was “I feel trapped in algorithmic echo chambers” mean 2.99 (SD 1.10), followed by “Algorithms impact my emotional state” mean 2.31 (SD 1.05), suggesting that while participants acknowledge the emotional and cognitive influence of algorithmic filtering, their active concern or resistance remains moderate. Other items, such as “I try to resist algorithmic influence” mean 2.10 (SD 1.02) and “I’m concerned about algorithmic manipulation” mean 2.00 (SD 1.00), received lower endorsement, indicating that the internalization of algorithmic impact is more cognitive than behavioral for most users.

Correlational analysis revealed several significant associations with PSMU as indexed by the CAGE-AID. The strongest relationship was observed for “I try to resist algorithmic influence” (ρ=0.155; *P*=.002), followed by “Algorithms reinforce my existing beliefs” (ρ=0.149; *P*=.003) and “Algorithms impact my emotional state” (ρ=0.139; *P*=.006). These results suggest that individuals who are more attuned to or concerned about algorithmic shaping of their feeds may also be more vulnerable to patterns of compulsive or dysregulated use. Interestingly, feeling trapped in echo chambers did not reach statistical significance (ρ=0.075; *P*=.14), possibly reflecting a broader but less emotionally salient experience.

Age-based subgroup analyses showed modest differences. Younger participants (aged 18-34 years) tended to report slightly higher algorithm awareness and concern, with average scores of approximately 2.00 to 2.13 for items such as “Algorithms influence what I see online” and “I’m concerned about algorithmic manipulation.” The oldest group (aged 65 years or older), however, reported lower levels of perceived influence mean 1.22 (SD 0.80) and concern mean 1.56 (SD 0.90),consistent with possible generational gaps in algorithmic literacy or engagement intensity. Nevertheless, feelings of being trapped in echo chambers remained relatively elevated across all age groups, suggesting that algorithmic filtering may be broadly recognized regardless of demographic background.

### Digital Detox Behaviors: Coping Strategies and Associations With Problematic Use

**Figure 6:**
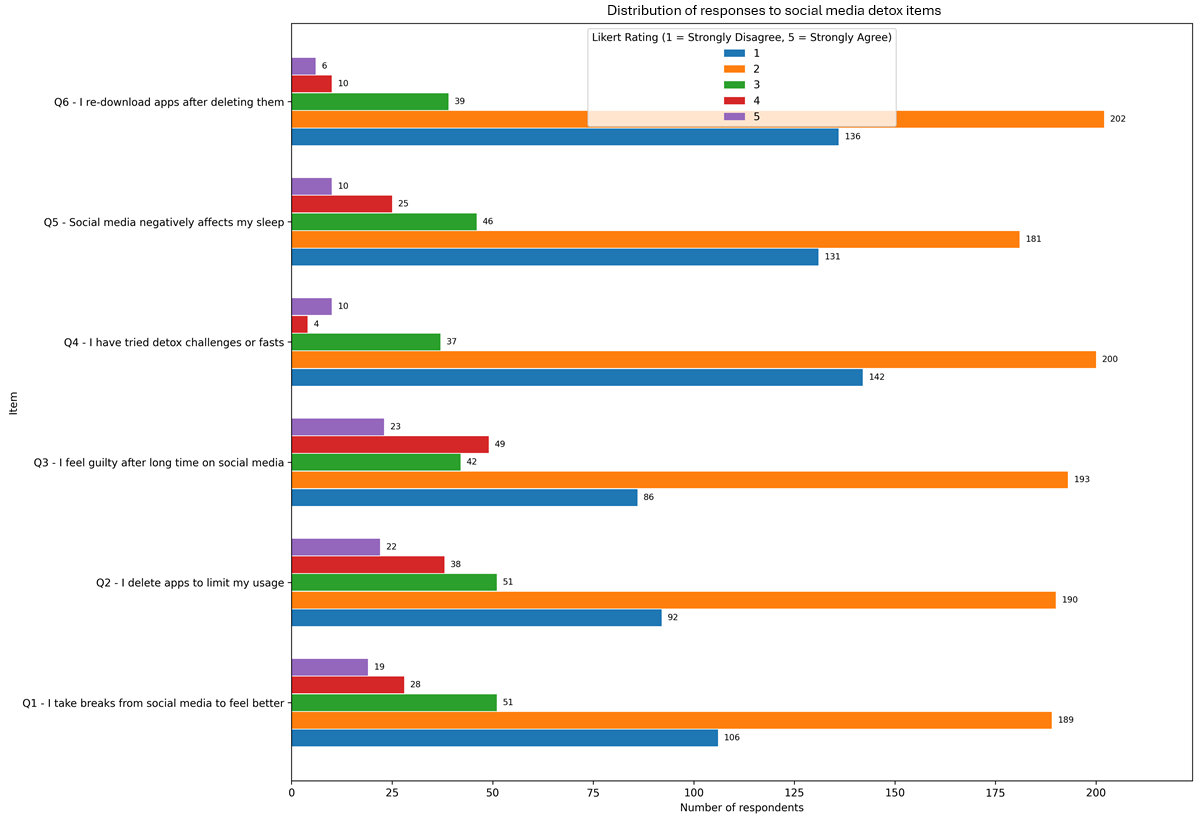
Mean endorsement (1-5 Likert scale) of digital detox behaviors among adult social media users in a cross-sectional online survey (8-week recruitment period).

Survey responses indicate that digital detox behaviors (such as taking breaks, deleting apps, or attempting challenges) are moderately common among participants and presented in Figure 6. The most frequently endorsed item was “I feel guilty after long time on social media” mean 2.31 (SD 1.05), followed by “I delete apps to limit my usage” mean 2.26 (SD 1.03) and “I take breaks from social media to feel better” mean 2.15 (SD 1.02; μ=2.15). These results suggest a notable level of self-awareness and adaptive attempts at behavioral regulation. Less frequently endorsed behaviors included “I try detox challenges or fasts” mean 1.83 (SD 0.95) and “I re-download apps after deleting them” (μ=1.99), which reflect more intermittent or ambivalent strategies.

Importantly, several detox behaviors showed statistically significant positive correlations with CAGE-AID positivity, suggesting a link between attempts to regulate use and potential problematic engagement. The strongest associations were observed for “I feel guilty after long time on social media” (ρ=0.200; *P*<.001) and “Social media negatively affects my sleep” (ρ=0.187; *P*<.001), underscoring emotional and physiological consequences of excessive use. Other significant associations included app deletion (ρ=0.159; *P*=.002) and taking breaks (ρ=0.138; *P*=.006). These results suggest that individuals engaging in detox behaviors may already recognize maladaptive patterns and are attempting to restore control, but with varying success.

Subgroup analysis by age revealed that younger users were more likely to engage in detox-related behaviors, particularly in deleting apps and expressing guilt. Mean scores for app deletion, guilt, and perceived sleep impacts all hovered around 2.25 to 2.31, while older adults consistently scored lower on these items. This age gradient suggests that younger individuals (potentially more embedded in digital environments) may also be more prone to cyclical disengagement strategies as a form of digital self-care or reactive control. These findings highlight a tension between awareness and effectiveness of digital detox strategies. While users, particularly younger ones, are actively attempting to regulate their consumption, the correlations with CAGE-AID positivity suggest that detox behaviors often arise in response to distress rather than prevention, reinforcing the need for proactive and sustainable digital well-being interventions.

### Nostalgia and Reminders: Emotional Reactions and Coping Mechanisms in the Digital Archive

**Figure 7:**
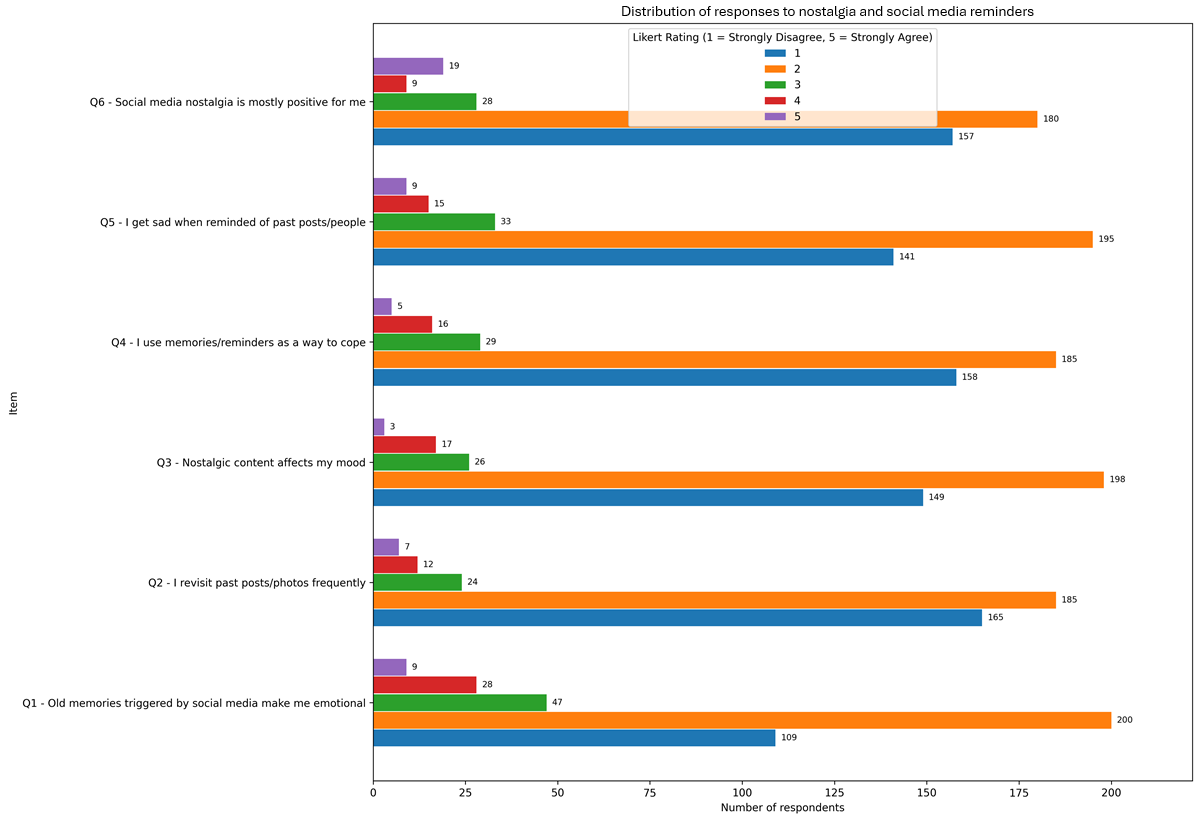
Mean endorsement (1-5 Likert scale) of nostalgia-related emotional responses to social media memories in adult users from a cross-sectional online study conducted over 8 weeks.

Participant responses to nostalgia-related items reveal that emotional engagement with past digital content is common but nuanced and are presented in Figure 7. The most endorsed items were “Old memories triggered by social media make me emotional” mean 2.05 (SD 1.00) and “I get sad when reminded of past posts or people” mean 1.87 (SD 0.95), suggesting that while the emotional impact is present, it is generally moderate. Other items, such as “I revisit past posts/photos frequently” mean 1.76 (SD 0.92) and “I use reminders as a way to cope” mean 1.79 (SD 0.93), highlight the potential dual function of nostalgic content—as both a stressor and a source of comfort. Interestingly, “Social media nostalgia is mostly positive” had the lowest endorsement mean 1.70 (SD 0.90), indicating a slightly negative or ambivalent emotional valence overall.

Correlational analyses showed modest but significant associations with CAGE-AID positivity, especially for items reflecting emotional reactivity. The strongest association was observed for “I use memories/reminders as a way to cope” (ρ=0.122; *P*=.02), followed by “Old memories make me emotional” (ρ=0.111; *P*=.03), and “Nostalgic content affects my mood” (ρ=0.104; *P*=.04). These findings suggest that users who respond more intensely to digital memories may also be more susceptible to PSMU (perhaps due to underlying affective vulnerabilities or rumination tendencies).

Age subgroup analysis showed that younger participants reported slightly higher emotional resonance with nostalgic content. In the subgroup with a positive CAGE-AID result, average scores hovered between 1.70 and 2.05 for all items. Although these means are modest, the consistent pattern across items supports the idea that social media nostalgia is a cross-cutting but variably experienced emotional phenomenon, more pronounced in individuals who engage frequently and emotionally with their digital history.

The responses of the survey are available in Multimedia Appendix 3.

### Translating Findings Into Practical Guidance

#### Overview

Although this study is cross-sectional in design and thus cannot infer causality, the patterns observed across nearly 400 participants offer meaningful insights into the psychological impact of everyday social media use. The consistency of associations between self-reported distress and specific digital behaviors highlights the need for more considerate digital well-being strategies. While further longitudinal and experimental research is required to validate these associations, the current findings offer a useful baseline from which preliminary guidance can be drawn. The following recommendations are intended for the general public and reflect trends observed in our dataset. They aim to support healthier and more mindful engagement with social media platforms, especially in an era where digital interactions are deeply embedded in daily life.

#### Be Mindful of How Social Media Affects Your Emotions

If you often feel drained, anxious, or self-critical after spending time online, it is worth pausing to reflect on which types of content or which platform features may be contributing to this. Recognizing these emotional patterns is a first step toward more conscious digital habits.

#### Set Gentle But Clear Boundaries Around Your Usage

Turn off unnecessary notifications, hide “read” receipts, or put your phone away during meals or bedtime. These small adjustments can significantly reduce feelings of pressure, improve sleep, and help restore a sense of control over your digital life.

#### Understand How Your Feed Is Shaped

Algorithms are designed to keep you engaged, often by prioritizing emotionally charged or repetitive content. Learning to recognize when your attention is being hijacked, and choosing to scroll more intentionally, can reduce stress and prevent compulsive behaviors.

#### Take Breaks Before You Burn Out

Rather than waiting until you feel overwhelmed, try scheduling short breaks from social media regularly. Unplugging periodically (even for a few hours) can reduce fatigue and help you reconnect with activities that restore your mental focus.

#### It Is Okay to Feel Conflicted About Social Media

Many people experience both positive and negative emotions online. Feeling nostalgic, left out, overwhelmed, or even manipulated by digital platforms is not a personal failing: it is a reflection of how these tools are designed. Being aware of this can help you navigate your digital life with more self-compassion and intention.

## Discussion

### Principal Results

In this study, we investigated the psychosocial effects of social media use among adults, focusing on how specific features of digital platforms relate to emotional well-being. One of the most important findings was the remarkably high proportion of respondents who screened positive on the adapted CAGE-AID tool for PSMU, particularly among younger adults, those with lower educational attainment, and those spending more time online. Emotional fatigue and empathic desensitization emerged as dominant experiences, with participants reporting avoidance of emotional content and feelings of overwhelm. Features embedded in messaging platforms (such as read receipts and typing indicators) were associated with elevated stress levels and compulsive checking behaviors. Respondents also expressed moderate internal conflict related to online self-presentation, with younger users more likely to curate idealized versions of themselves. Engagement-driven behaviors such as comparing metrics or aspiring to virality were modestly endorsed but nonetheless correlated with problematic use. Finally, awareness of algorithmic filtering and digital detox attempts reflected a self-perceived need for behavioral regulation, while nostalgic digital content elicited mixed emotional reactions, serving both as sources of comfort and distress. These findings highlight the complex interplay between platform design, individual vulnerabilities, and psychological strain.

### Comparison With Prior Work

The association between emotional exhaustion and PSMU aligns with existing work suggesting that constant exposure to emotionally charged or traumatic content can lead to “compassion fatigue” or emotional burnout [31,32]. Emotional avoidance and decreased empathy, as reported in our sample, echo findings from Elhai [33], who documented emotional desensitization among heavy users. These patterns may be exacerbated by the algorithmic amplification of emotionally provocative content, which can prolong emotional arousal and disrupt emotional regulation [34]. Similarly, our findings on silent stressors resonate with studies showing that even minor interface cues can provoke anxiety, compulsive checking, and performance-related pressure [35]. These stressors, often dismissed as trivial, may accumulate to create chronic low-grade distress, particularly in users already prone to digital overuse or social sensitivity.

Our observations regarding online identity support prior research emphasizing the psychological toll of managing multiple selves online. This is similar to the findings of Marwick and Boyd [36] who described the “context collapse” effect, wherein individuals navigate overlapping social audiences and expectations, leading to increased self-monitoring and anxiety. Younger participants in this study showed greater pressure to idealize their online image, consistent with the literature that adolescents and young adults are particularly vulnerable to appearance-based social comparisons [37,38]. Likewise, engagement with virality (though not universally endorsed) was linked to greater emotional investment in likes, shares, and public validation. This is in line with Yang and Robinson’s [39] work on “quantified self-worth,” wherein digital metrics become a proxy for self-esteem. Even among adults, our findings suggest that the aspiration to “go viral” or modify content for maximum reach is not uncommon and may contribute to a cycle of compulsive posting and performance anxiety.

The study also highlights algorithmic awareness as a psychosocial construct in its own right. Although participants expressed feeling emotionally affected by curated feeds and filter bubbles, only a minority reported actively resisting algorithmic influence suggesting a cognitive dissonance between perceived manipulation and behavioral response. This reflects Roth’s [40] notion of algorithmic disturbances where users feel that digital environments shape their behaviors beyond their control. Moreover, our findings on digital detox behaviors provide important insight into the limits of self-regulation. While many participants reported deleting apps, taking breaks, or experiencing guilt, these behaviors were also associated with higher modified CAGE-AID scores, which implies that detox efforts may often be reactive, triggered by distress rather than preventive. This echoes observations by Roberts and David [41], who found that digital disconnection is frequently short-lived and emotionally ambivalent. Lastly, the role of digital nostalgia as both a coping mechanism and a source of sadness has been underexplored in the literature. Our findings suggest that reminders of past posts and memories can evoke emotional turbulence, particularly in individuals already prone to compulsive engagement, and this should be further studied.

### Limitations

Amongst its limitations, the cross-sectional nature of the design precludes causal inferences, and the reliance on self-reported data may introduce biases such as social desirability or recall distortion. The adapted CAGE-AID, while grounded in conceptual analogies to behavioral addiction, has not yet been psychometrically validated for digital contexts, which limits its diagnostic utility. Additionally, the sample was not representative of the general population, with an overrepresentation of younger, urban, and educated respondents. These factors limit generalizability and suggest the need for replication in more diverse cohorts.

### Conclusions

This study offers a portrait of the psychological impacts of social media use among adults, emphasizing the interplay between digital platform features, emotional regulation, and self-perception. By investigating dimensions such as empathic fatigue, identity fragmentation, algorithmic distress, and digital nostalgia, the findings move beyond simplistic metrics like screen time to reveal the complex emotional and cognitive terrain of online engagement. A significant proportion of participants screened positive for PSMU, particularly among younger and more digitally immersed users. Subtle design elements, such as read receipts, algorithmic feeds, and visibility metrics, emerged as meaningful contributors to digital stress and are often overlooked in public discourse. This underscores the need to reconceptualize digital well-being not just as a function of usage duration, but of platform architecture and emotional exposure. As social media becomes increasingly embedded in daily life, mental health professionals, educators, and platform designers must collaboratively develop strategies to mitigate its more insidious effects. Future research should build on these findings to create validated tools for digital distress screening and intervention. In conclusion, designing healthier digital habits will require a more empathetic and evidence-informed understanding of how people navigate these emotionally charged environments.

## Data Availability

All data generated or analyzed during this study are included in this published article as Multimedia Appendix 3.

## Appendix

Multimedia Appendix 1 STROBE checklist.

Multimedia Appendix 2 French and English full questionnaires.

Multimedia Appendix 3 Full dataset of the answers of the respondents.

## Abbreviations

CAGE-AID: Cut down, Annoyed, Guilty, Eye-opener–Adapted to Include Drugs
PSMU: problematic social media use
STROBE: Strengthening the Reporting of Observational Studies in Epidemiology
