# Supplementary material for: Emotional and Psychosocial Correlates of Problematic Social Media Use Among Adults: Cross-Sectional Study
Source: JMIR Form Res. 2026 Apr 1;10:e82098. doi: 10.2196/82098 (PMC13085987; doi:10.2196/82098)
Supplement: Multimedia Appendix 2 [file formative_v10i1e82098_app2.pdf]

Age: *

Choose one of the following answers
Please choose **only one** of the following:

- ◯ 18-24
- ◯ 25-34
- ◯ 35-44
- ◯ 45-54
- ◯ 55-64
- ◯ 65 and above

Gender: *

Choose one of the following answers
Please choose **only one** of the following:

- ◯ Female
- ◯ Male
- ◯ Non-binary
- ◯ Prefer not to answer
- ◯ Other

Country of residence: *

Please write your answer here:

Living Environment: *

Choose one of the following answers
Please choose **only one** of the following:

- Urban (city)
- Suburban (near a big city)
- Rural (countryside)

Employment status: *

Choose one of the following answers
Please choose **only one** of the following:

- Student
- Employed full-time (36 + hours / week)
- Employed part-time
- Unemployed
- Self-employed/Freelancer
- Retired
- Other

Level of Education: *

Choose one of the following answers
Please choose **only one** of the following:

- High School or less
- Vocational training or Diploma
- Undergraduate Degree
- Graduate or Professional Degree (Master's, PhD, MD, etc.)
- Other

Estimated Time Spent Daily on Social Media: *

Choose one of the following answers
Please choose **only one** of the following:

- Less than 1 hour/day
- 1-2 hours/day
- 3-4 hours/day
- 5-6 hours/day
- 7 hours or more/day

Social Media Usage:

By “social media,” we mean any online discussion platform—Facebook, Instagram, Snapchat, Reddit, TikTok, X, and so on.

Please answer Yes or No to the following: *

Please choose the appropriate response for each item:

	Yes	No
**Have you ever felt you should cut down your social media usage?**	◯	◯
**Do you get annoyed or irritated when others criticize your social media habits?**	◯	◯
**Have you ever felt guilty or regretful about how much time you spend on social media?**	◯	◯
**Do you check social media immediately upon waking up to feel connected or less anxious?**	◯	◯

Digital Empathy Fatigue

Psychological exhaustion caused by the continual demand for empathy in online interactions, which gradually diminishes one's capacity to respond emotionally to digital content.

Please respond using the following scale: (1 = Strongly disagree, 5 = Strongly agree):

*

Please choose the appropriate response for each item:

	1	2	3	4	5
**Seeing emotionally heavy posts makes me feel emotionally exhausted.**	○	○	○	○	○
**I feel less compassionate after prolonged emotional content exposure.**	○	○	○	○	○
**Constant emotional struggles online decrease my empathy.**	○	○	○	○	○
**I avoid emotional engagement due to overwhelm.**	○	○	○	○	○
**Repeated online tragedies make me numb offline.**	○	○	○	○	○
**Social media made me indifferent to emotional difficulties.**	○	○	○	○	○

Describe a situation where online emotional content affected your empathy.

Please write your answer here:

What coping strategies do you use for emotional exhaustion from social media?

Please write your answer here:

Silent Stressors (Read Receipts)

Silent factors: read receipts that, without any visible interaction, inform the sender a message has been seen

Please respond using the following scale: (1 = Strongly disagree, 5 = Strongly agree): *

Please choose the appropriate response for each item:

	1	2	3	4	5
**I have anxiety when messages are read without immediate reply.**	○	○	○	○	○
**Seeing the "Typing…" indicator causes me stress.**	○	○	○	○	○
**I delay opening messages due to read receipts.**	○	○	○	○	○
**Read receipts negatively affect my mood.**	○	○	○	○	○
**Overthinking interactions due to "seen" indicators.**	○	○	○	○	○
**Pressure to respond quickly due to "seen" indicators.**	○	○	○	○	○

How do read receipts impact your mental well-being?

Please write your answer here:

Share an experience of anxiety related to messaging indicators.

Please write your answer here:

Identity Fragmentation

Identity fragmentation: the tendency to present different facets of oneself across multiple social platforms, creating partial and sometimes inconsistent versions of the same individual.

Please respond using the following scale: (1 = Strongly disagree, 5 = Strongly agree): *

Please choose the appropriate response for each item:

	1	2	3	4	5
**Habing multiple online profiles feel mentally exhausting.**	○	○	○	○	○
**I have anxiety managing my online personas.**	○	○	○	○	○
**My online identities make me question my true self.**	○	○	○	○	○
**I feel disconnected from myself managing online personas.**	○	○	○	○	○
**My online identities negatively impact offline mental health.**	○	○	○	○	○
**I worry about social misunderstandings due to my online identities.**	○	○	○	○	○

How does managing multiple identities affect your mental health?

Please write your answer here:

Describe when your online identity conflicted with your real-life self.

Please write your answer here:

Virality Pressure (being popular online)

Virality pressure (online popularity): the sense of compulsion to create or share content that could go viral, fueling a competitive race for visibility and engagement among social-media users.

Please respond using the following scale: (1 = Strongly disagree, 5 = Strongly agree): *

Please choose the appropriate response for each item:

	1	2	3	4	5
**Going viral excites but makes me anxious.**	○	○	○	○	○
**I stress replicating my past social media success.**	○	○	○	○	○
**My mood fluctuates with online attention**	○	○	○	○	○
**Less reactions negatively impact mental health.**	○	○	○	○	○
**I feel pressure to consistently create popular content.**	○	○	○	○	○
**Sudden online popularity significantly impacted my well-being.**	○	○	○	○	○

How has virality (online popularity) affected your mental health?

Please write your answer here:

Accoridng to you, what is the emotional impact of maintaining online popularity?

Please write your answer here:

Algorithm-Induced Mood Swings

Please respond using the following scale: (1 = Strongly disagree, 5 = Strongly agree): *

Please choose the appropriate response for each item:

	1	2	3	4	5
**Algorithms remove my emotional control over social media.**	○	○	○	○	○
**I feel frustrated when algorithms show unwanted content.**	○	○	○	○	○
**My mood is influenced by unpredictability of feed.**	○	○	○	○	○
**Algorithm control negatively affects mental health.**	○	○	○	○	○
**Algorithms increase helplessness or irritation.**	○	○	○	○	○
**Algorithm-curated content causes emotional fluctuations.**	○	○	○	○	○

Describe how social media algorithms (for example: what is displayed in reels, or newsfeed) impact your mood.

Please write your answer here:

What are your feelings about algorithm-controlled content exposure?

Please write your answer here:

Social Media Detox (stopping for a few hours, days, weeks) Paradox

Please respond using the following scale: (1 = Strongly disagree, 5 = Strongly agree): *

Please choose the appropriate response for each item:

	1	2	3	4	5
**Social media breaks initially increase anxiety.**	○	○	○	○	○
**I feel isolated during social media breaks.**	○	○	○	○	○
**I experience "FOMO : fear of missing out" when offline.**	○	○	○	○	○
**Social media breaks negatively impacts mood or relationships.**	○	○	○	○	○
**It is difficult to stay offline due to anxiety.**	○	○	○	○	○
**Social media breaks experiences are less positive than expected.**	○	○	○	○	○

What are your personnal experiences during social media breaks (eg.: is it good for you, bad, etc.)?

Please write your answer here:

What were your unexpected emotions during social media breaks?

Please write your answer here:

Social Media Nostalgia Anxiety

Anxiety triggered by digital reminders and memories (old posts, “memories”) that confront the user with an idealized version of their past.

Please respond using the following scale: (1 = Strongly disagree, 5 = Strongly agree): *

Please choose the appropriate response for each item:

	1	2	3	4	5
**Old memories online cause sadness or anxiety.**	○	○	○	○	○
**Dread notifications of memories.**	○	○	○	○	○
**Past events online negatively affect mood.**	○	○	○	○	○
**Nostalgia features cause emotional distress.**	○	○	○	○	○
**Memories lead to rumination or regret.**	○	○	○	○	○
**Wish to disable nostalgia reminders.**	○	○	○	○	○

Please provide an example (if any) of an emotional response triggered by social media memories?

Please write your answer here:

What impact do reminders of past events (e.g., an old party, a graduation, etc.) have on your emotional well-being?

Please write your answer here:

End of the Questionnaire

If you would like to be kept informed about the results of this study, please provide your email address on the next page (your email will not be linked to your responses).

Email:

Please write your answer here:

Thank you for participating!

Submit your survey.
Thank you for completing this survey.

Votre âge: *

Veuillez sélectionner une réponse ci-dessous.
Veuillez sélectionner une seule des propositions suivantes :

- ◯ 18-24
- ◯ 25-34
- ◯ 35-44
- ◯ 45-54
- ◯ 55-64
- ◯ 65 et plus

Genre: *

Veuillez sélectionner une réponse ci-dessous.
Veuillez sélectionner une seule des propositions suivantes :

- ◯ Féminin
- ◯ Masculin
- ◯ Non-binaire
- ◯ Préfère ne pas répondre
- ◯ Autre

Pays de résidence: *

Veuillez écrire votre réponse ici :

Milieu de vie: *

Veuillez sélectionner une réponse ci-dessous.
Veuillez sélectionner une seule des propositions suivantes :

- ◯ Urbain (ville)
- ◯ Banlieue (bordure d'une grande ville)
- ◯ Rural (campagne)

Emploi actuel: *

Veuillez sélectionner une réponse ci-dessous.
Veuillez sélectionner une seule des propositions suivantes :

- ◯ Étudiant
- ◯ Emploi temps-plein (36 heures par semaine)
- ◯ Emploi à temps-partiel (moins de 36 heures par semaine)
- ◯ Sans emploi (ni aux études)
- ◯ Travailleur autonome
- ◯ Retraité
- ◯ Autre

Plus haut niveau d'éducation: *

Veuillez sélectionner une réponse ci-dessous.
Veuillez sélectionner une seule des propositions suivantes :

- ◯ Secondaire ou inférieur
- ◯ Formation professionnelle ou diplôme technique
- ◯ Diplôme universitaire de premier cycle
- ◯ Diplôme universitaire de deuxième cycle ou professionnel (Maîtrise, Doctorat, Médecine, etc.)
- ◯ Autre

Temps quotidien estimé passé sur les médias sociaux : *

Veuillez sélectionner une réponse ci-dessous.
Veuillez sélectionner une seule des propositions suivantes :

- ◯ Moins d'une heure/jour
- ◯ 1-2 heures/jour
- ◯ 3-4 heures/jour
- ◯ 5-6 heures/jour
- ◯ 7 heures ou plus/jour

Utilisation des médias sociaux

Par médias sociaux, nous entendons toutes plateformes de discussion en ligne : Facebook, Instagram, Snapchat, Reddit, TikTok, X, etc.

Veuillez répondre par Oui ou Non aux questions suivantes : *

Choisissez la réponse appropriée pour chaque élément :

	Oui	Non
**Avez-vous déjà ressenti le besoin de réduire votre utilisation des médias sociaux ?**	○	○
**Êtes-vous agacé(e) ou irrité(e) lorsque d'autres critiquent vos habitudes sur les médias sociaux ?**	○	○
**Avez-vous déjà ressenti de la culpabilité ou du regret concernant le temps passé sur les médias sociaux ?**	○	○
**Vérifiez-vous les médias sociaux immédiatement au réveil pour vous sentir connecté(e) ou moins anxieux(se) ?**	○	○

Fatigue de l’empathie numérique

Épuisement psychologique causé par la sollicitation continue d’empathie dans les interactions en ligne, qui réduit progressivement la capacité à répondre émotionnellement aux contenus numériques.

Merci de répondre selon l'échelle : (1= Pas du tout d'accord, 5= Tout à fait d'accord): *

Choisissez la réponse appropriée pour chaque élément :

	1	2	3	4	5
**Voir des publications émotionnelles m'épuise émotionnellement.**	○	○	○	○	○
**Je suis moins compatissant(e) après exposition prolongée aux contenus émotionnels.**	○	○	○	○	○
**Les difficultés émotionnelles constantes en ligne diminuent mon empathie.**	○	○	○	○	○
**J'évite l'engagement émotionnel car c’est accablant.**	○	○	○	○	○
**Les tragédies répétées en ligne me rendent insensible hors ligne.**	○	○	○	○	○
**Les médias sociaux m'ont rendu(e) indifférent(e) aux difficultés émotionnelles.**	○	○	○	○	○

Décrivez une situation où un contenu émotionnel en ligne a affecté votre empathie.

Veuillez écrire votre réponse ici :

Quelles stratégies utilisez-vous contre l’épuisement émotionnel lié aux médias sociaux ?

Veuillez écrire votre réponse ici :

Facteurs silencieux (Confirmations de lecture)

Facteurs silencieux : confirmations de lecture qui, sans interaction visible, informent l’expéditeur qu’un message a été vu.

Merci de répondre selon l'échelle: (1= Pas du tout d'accord, 5= Tout à fait d'accord):

*

Choisissez la réponse appropriée pour chaque élément :

	1	2	3	4	5
**Je ressens de l'anxiété si messages lus sans réponse immédiate.**	○	○	○	○	○
**L'indicateur « écrit/typing… » me cause du stress.**	○	○	○	○	○
**Je retarde l'ouverture des messages à cause des confirmations.**	○	○	○	○	○
**Les confirmations de lecture affectent négativement mon humeur.**	○	○	○	○	○
**Je réfléchis trop aux interactions à cause des indicateurs « vu ».**	○	○	○	○	○
**Je ressens une pression à répondre rapidement à cause des indicateurs « vu ».**	○	○	○	○	○

Quel est l'impact des confirmations de lecture sur votre santé mentale ?

Veuillez écrire votre réponse ici :

Pouvez-vous nous partager une expérience en lien avec les indicateurs des messages (par exemple, que ce soit écrit vu ou ''en train d'écrire'').

Veuillez écrire votre réponse ici :

Fragmentation de l’identité

Fragmentation de l’identité : tendance à dissocier et afficher différents aspects de soi sur plusieurs plateformes sociales

Merci de répondre selon l'échelle: (1= Pas du tout d'accord, 5= Tout à fait d'accord) *

Choisissez la réponse appropriée pour chaque élément :

	1	2	3	4	5
**Gérer plusieurs profils m'épuise mentalement.**	○	○	○	○	○
**Je ressens de l'anxiété à gérer mes différentes personnalités en ligne.**	○	○	○	○	○
**Mes identités en ligne me font douter de mon vrai moi.**	○	○	○	○	○
**Je me sens déconnecté(e) de moi-même à gérer des personnalités en ligne.**	○	○	○	○	○
**Mes identités en ligne affectent négativement ma santé mentale hors ligne.**	○	○	○	○	○
**Je crains d’être mal compris(e) à cause de mes identités en ligne.**	○	○	○	○	○

Comment vos multiples identités en ligne affectent-elles votre santé mentale ?

Veuillez écrire votre réponse ici :

Décrivez un exemple de quand votre identité en ligne était en conflit avec votre vrai ''moi'' (par exemple, un moment où vous aviez l'impression que votre identité en ligne ne réflétait par vraiment qui vous êtes).

Veuillez écrire votre réponse ici :

Pression de la viralité (être populaire en ligne)

Pression de la viralité (être viral en ligne) : sentiment d’obligation à produire ou partager des contenus susceptibles de devenir viraux, générant une course à la visibilité et à l’engagement des utilisateurs de médias sociaux.

Merci de répondre selon l'échelle: (1= Pas du tout d'accord, 5= Tout à fait d'accord) *

Choisissez la réponse appropriée pour chaque élément :

	1	2	3	4	5
**Le fait de devenir viral (populaire) m’excite mais me rend anxieux.**	○	○	○	○	○
**Je ressens du stress à vouloir reproduire mes succès passés sur les réseaux sociaux.**	○	○	○	○	○
**Mon humeur fluctue en fonction de l’attention que je reçois en ligne.**	○	○	○	○	○
**Un manque de réactions a un impact négatif sur ma santé mentale.**	○	○	○	○	○
**Je ressens une pression à créer constamment du contenu populaire.**	○	○	○	○	○
**Une popularité soudaine a significativement affecté mon bien-être.**	○	○	○	○	○

Comment la viralité (popularité en ligne) a-t-elle affecté votre santé mentale ?

Veuillez écrire votre réponse ici :

Quel est l'impact émotionnel du maintien d'une popularité en ligne selon-vous?

Veuillez écrire votre réponse ici :

Sautes d'humeur induits par les algorithmes

Merci de répondre selon l'échelle: (1= Pas du tout d'accord, 5= Tout à fait d'accord)
*

Choisissez la réponse appropriée pour chaque élément :

	1	2	3	4	5
**Les algorithmes me font perdre le contrôle (de mes émotions) sur les réseaux sociaux.**	○	○	○	○	○
**Je ressens de la frustration lorsque les algorithmes me montrent du contenu non désiré.**	○	○	○	○	○
**Mon humeur est influencée par l’imprévisibilité du fil d’actualité.**	○	○	○	○	○
**Le contrôle algorithmique affecte négativement ma santé mentale.**	○	○	○	○	○
**Les algorithmes augmentent mon sentiment d’impuissance ou d’irritabilité.**	○	○	○	○	○
**Le contenu sélectionné par les algorithmes entraîne des fluctuations émotionnelles.**	○	○	○	○	○

Décrivez comment les algorithmes (par exemple les vidéos ou informations choisies pour vous par le média social) influencent votre humeur.

Veuillez écrire votre réponse ici :

Que ressentez-vous face à l'exposition au contenu contrôlé/affiché par les algorithmes de façon général?

Veuillez écrire votre réponse ici :

Le paradoxe de la détox (arrêt de quelques heures, jours, semaines) des réseaux sociaux

Merci de répondre selon l'échelle: (1= Pas du tout d'accord, 5= Tout à fait d'accord) *

Choisissez la réponse appropriée pour chaque élément :

	1	2	3	4	5
**Les pauses des réseaux sociaux augmentent d'abord mon anxiété.**	○	○	○	○	○
**Je me sens isolé pendant une pause des réseaux sociaux.**	○	○	○	○	○
**Je ressens du FOMO (peur de manquer quelque chose) quand je suis hors ligne.**	○	○	○	○	○
**Une pause des réseaux sociaux affecte négativement mon humeur ou mes relations.**	○	○	○	○	○
**Il m'est difficile de rester hors ligne à cause de l'anxiété.**	○	○	○	○	○
**Les expériences de pauses des réseaux sociaux sont moins positives que ce que j'attendais.**	○	○	○	○	○

Quelles ont été vos expériences personnelles durant une pause des réseaux sociaux (par exemple, est-ce bon pour vous, mauvais, etc.)?

Veuillez écrire votre réponse ici :

Quelles émotions inattendues avez-vous ressenties pendant ces pauses ?

Veuillez écrire votre réponse ici :

L'anxiété nostalgique liée aux réseaux sociaux

Anxiété suscitée par les rappels et souvenirs numériques (anciennes publications, « memories ») qui confrontent l'utilisateur à une version idéalisée de son passé

Merci de répondre selon l'échelle: (1= Pas du tout d'accord, 5= Tout à fait d'accord) *

Choisissez la réponse appropriée pour chaque élément :

	1	2	3	4	5
**Les anciens souvenirs en ligne me rendent triste ou anxieux.**	○	○	○	○	○
**Je redoute les notifications de souvenirs.**	○	○	○	○	○
**Les événements passés visibles en ligne affectent négativement mon humeur.**	○	○	○	○	○
**Les fonctions de nostalgie génèrent une détresse émotionnelle.**	○	○	○	○	○
**Les souvenirs entraînent de la rumination ou du regret.**	○	○	○	○	○
**Je souhaiterais désactiver les rappels nostalgiques.**	○	○	○	○	○

Quelle réponse émotionnelle les souvenirs sur les réseaux sociaux déclenchent-ils chez vous ?

Veuillez écrire votre réponse ici :

Quel est l'impact des rappels d'événements passés sur votre bien-être émotionnel (ex.: une ancienne fête, une graduation, etc.)?

Veuillez écrire votre réponse ici :

Fin du questionnaire

Si vous souhaitez être tenu.es au courant des résultats de cette étude, merci de nous laissez votre courriel à la page suivante (votre courriel ne serait pas colligé avec vos réponses).

Courriel:

Veuillez écrire votre réponse ici :

Envoyer votre questionnaire.

Merci d’avoir complété ce questionnaire.
